# Supplementary material for: Heart Rate Variability in Hyperthyroidism: A Systematic Review and Meta-Analysis
Source: Int J Environ Res Public Health. 2022 Mar 18;19(6):3606. doi: 10.3390/ijerph19063606 (PMC8949365; doi:10.3390/ijerph19063606)
Supplement: Supplementary file 1 [file ijerph-19-03606-s001.zip › ijerph-1621358-supplementary.pdf]

Figure S1. Details for the search strategy used within each database

Pubmed
(hyperthyroidism[TW] OR hyperthyroid[TW]) **AND** ("heart rate variability"[TW] OR HRV[TW])
Filter Language = 0
Filter Dates = 0
https://pubmed.ncbi.nlm.nih.gov/?term=%28hyperthyroidism%5BTW%5D+OR+hyperthyroid%5BTW%5D%29+AND+%28%22heart+rate+variability%22%5BTW%5D+OR+HRV%5BTW%5D%29

= 35 articles

Cochrane Library
(“hyperthyroidism” OR "hyperthyroid") **AND** ("heart rate variability" OR "HRV")
In All Text
Filter Language = 0
Filter Dates = 0
https://www.cochranelibrary.com/search?p_p_id=scolarissearchresultsportlet_WAR_scolarissearchresults&p_p_lifecycle=0&_scolarissearchresultsportlet_WAR_scolarissearchresults_searchType=basic&_scolarissearchresultsportlet_WAR_scolarissearchresults_searchBy=6&_scolarissearchresultsportlet_WAR_scolarissearchresults_searchText=%28%E2%80%9Chyperthyroidism%E2%80%9D+OR+%22hyperthyroid%22%29++AND++%28%22heart+rate+variability%22+OR+%22HRV%22%29

= 8 articles

Embase
('hyperthyroidism'/exp OR 'hyperthyroid'/exp) **AND** ('heart rate variability'/exp OR ‘HRV'/exp)
Filter Language = 0
Filter Dates = 0

= 76 articles

Google Scholar
(“hyperthyroidism” OR "hyperthyroid") **AND** ("heart rate variability" OR "HRV")
Filter Language = 0
Filter Dates = 0
In Journal articles
https://scholar.google.fr/scholar?q=(%E2%80%9Chyperthyroidism%E2%80%9D+OR+%22hyperthyroid%22)+++AND+++(%22heart+rate+variability%22+OR+%22HRV%22)&hl=fr&as_sdt=0,5&as_vis=1&as_rr=1

= 519 articles

Figure S2. Quality of included studies

Methodological quality of included studies using the SIGN checklist

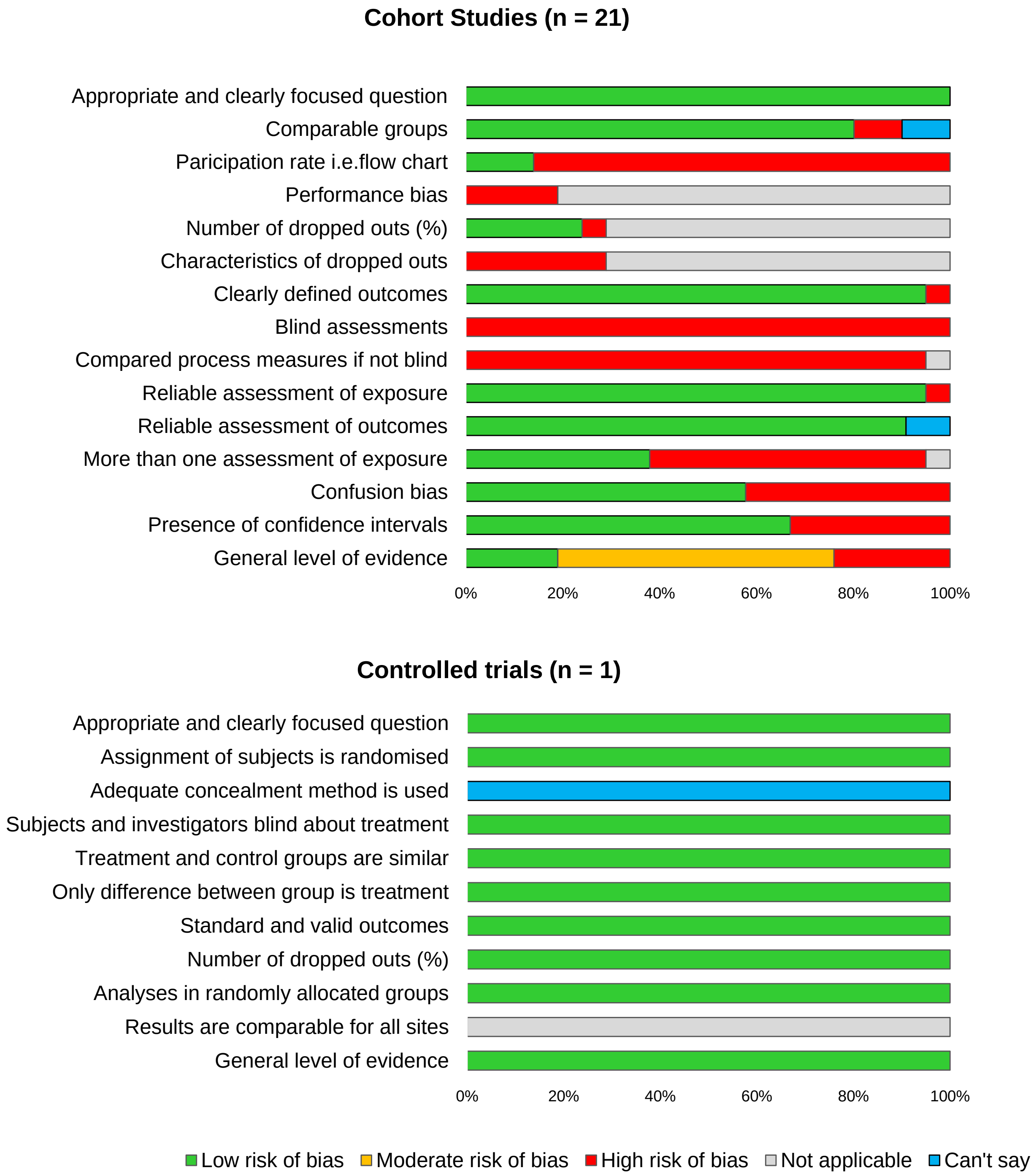

SIGN: Scottish Intercollegiate Guidelines Network

Methodological quality of included studies using the SIGN checklist, by study

Legend	
+	Yes
NA	Not applicable
?	Can't say
-	No
-	Low quality
+	Acceptable
++	High quality

	**Section 1: Studies conception**														**Section 2: Global evaluation of the paper**			
	Appropriate and clearly focused question	Comparable groups	Participation rate i.e flow chart	Performance bias	Number of dropped outs (%)	Characteristics of dropped outs	Clearly defined outcomes	Blind assessments	Compared process measures of not blind	Reliable assessment of exposure	Reliable assessment of outcomes	More than one assessment of exposure	Confusion bias	Presence of confidence intervals	Minimise the risk of bias and to establish a causal relationship	Clear evidence of an association between exposure and outcome	Results directly applicable	**General level of evidence**
Burggraaf 2001	+	-	-	-	+	-	+	-	+	+	+	-	-	+	+	+	+	+
Cacciatori 1996	+	+	-	NA	+	-	+	-	-	+	+	+	-	+	+	+	+	+
Cai 2018	+	+	-	NA	+	-	+	-	-	+	+	+	+	+	++	+	+	+
Chen 2006	+	+	-	NA	+	-	+	-	-	+	+	+	+	-	+	+	+	+
Chen 2007	+	+	-	-	NA	NA	+	-	-	+	+	-	-	+	+	+	+	+
Chen 2010	+	+	-	-	NA	NA	+	-	-	+	+	-	+	+	+	+	+	+
Falcone 2014	+	+	+	NA	NA	NA	+	-	-	+	+	+	+	-	+	+	+	+
Galetta 2009	+	+	-	NA	NA	NA	+	-	-	+	+	-	+	-	+	+	+	+
Goichot 2004	+	-	-	NA	NA	NA	+	-	-	+	+	+	-	-	+	+	+	-
Kabir 2009	+	+	-	-	NA	NA	+	-	-	+	+	+	+	+	+	+	+	+
Karthik 2009	+	+	-	NA	NA	NA	+	-	-	+	+	-	+	+	+	+	+	+
Mavai 2018	+	+	-	NA	-	-	+	-	-	+	+	-	+	+	+	+	+	+
Ngassam 2018	+	?	-	NA	+	-	+	-	-	+	+	+	-	-	+	+	+	-
Osman 2004	+	+	+	NA	NA	NA	+	-	-	+	+	+	-	+	+	+	+	+
Peixoto de Miranda 2018	+	+	+	NA	NA	NA	+	-	-	+	+	NA	+	+	+	+	+	+
Petretta 2001	+	+	-	NA	NA	NA	+	-	-	+	+	-	+	+	+	+	+	+
Pitzalis 1998	+	?	-	NA	NA	NA	+	-	-	+	+	-	-	+	+	+	+	-
Portella 2007	+	+	-	NA	NA	NA	+	-	-	+	+	-	?	+	+	+	+	+
Ramanathan 2014	+	+	-	NA	NA	NA	+	-	NA	+	+	-	+	-	+	+	+	+
Tobaldini 2008	+	+	-	NA	NA	NA	-	-	-	-	+	-	-	+	-	+	+	-
Tudoran 2019	+	+	-	NA	NA	NA	+	-	-	+	+	-	?	-	-	-	-	-

Cohort studies

Legend	
+ (green)	Yes
NA	Not applicable
? (yellow)	Can't say
- (red)	No
-	Low quality
+	Acceptable
++	High quality

	Selection bias				Comparability bias		Outcome bias				
	Appropriate and clearly focused question	Assignement of subjects is randomised	Adequate concealment method is used	Subjects and investigators blind about treatment	Treatment and control groups are similar	Only difference between group is treatment	Standard and valid outcomes	Number of dropped outs (%)	Analyses in randomly allocated groups	Results are comparable for all sites	**General level of evidence**
Eustatia-Rutten 2008	+	+	?	+	+	+	+	+	+	NA	+

<u>Randomized controlled studies</u>

Methodology Checklist 3: Cohort studies

S I G N

Study identification (*Include author, title, year of publication, journal title, pages*)

Guideline topic:	Key Question No:	Reviewer:

Before completing this checklist, consider:

1. Is the paper really a cohort study? If in doubt, check the study design algorithm available from SIGN and make sure you have the correct checklist.
2. Is the paper relevant to key question? Analyse using PICO (Patient or Population Intervention Comparison Outcome). IF NO REJECT (give reason below). IF YES complete the checklist..

Reason for rejection: 1. Paper not relevant to key question □ 2. Other reason □ (please specify):

Please note that a retrospective study (ie a database or chart study) cannot be rated higher than +.

Section 1: Internal validity

In a well conducted cohort study:		***Does this study do it?***
1.1	The study addresses an appropriate and clearly focused question	Yes □ No □ Can't say □
SELECTION OF SUBJECTS		
1.2	The two groups being studied are selected from source populations that are comparable in all respects other than the factor under investigation.	Yes □ No □ Can't say □ Does not apply □
1.3	The study indicates how many of the people asked to take part did so, in each of the groups being studied.	Yes □ No □ Does not apply □
1.4	The likelihood that some eligible subjects might have the outcome at the time of enrolment is assessed and taken into account in the analysis.	Yes □ No □ Can't say □ Does not apply □
1.5	What percentage of individuals or clusters recruited into each arm of the study dropped out before the study was completed.	
1.6	Comparison is made between full participants and those lost to follow up, by exposure status.	Yes □ No □ Can't say □ Does not apply □

ASSESSMENT		
1.7	The outcomes are clearly defined.	Yes □ No □<br>Can’t say □
1.8	The assessment of outcome is made blind to exposure status. If the study is retrospective this may not be applicable.	Yes □ No □<br>Can’t say □ Does not apply □
1.9	Where blinding was not possible, there is some recognition that knowledge of exposure status could have influenced the assessment of outcome.	Yes □ No □<br>Can’t say □ □
1.10	The method of assessment of exposure is reliable.	Yes □ No □<br>Can’t say □
1.11	Evidence from other sources is used to demonstrate that the method of outcome assessment is valid and reliable.	Yes □ No □<br>Can’t say □ Does not apply□
1.12	Exposure level or prognostic factor is assessed more than once.	Yes □ No □<br>Can’t say □ Does not apply □
CONFOUNDING		
1.13	The main potential confounders are identified and taken into account in the design and analysis.	Yes □ No □<br>Can’t say □
STATISTICAL ANALYSIS		
1.14	Have confidence intervals been provided?	Yes □ No □
**SECTION 2: OVERALL ASSESSMENT OF THE STUDY**		
2.1	How well was the study done to minimise the risk of bias or confounding?	High quality (++) □<br>Acceptable (+) □<br>Unacceptable – reject 0
2.2	Taking into account clinical considerations, your evaluation of the methodology used, and the statistical power of the study, do you think there is clear evidence of an association between exposure and outcome?	Yes ☐ No ☐<br>Can’t say ☐
2.3	Are the results of this study directly applicable to the patient group targeted in this guideline?	Yes □ No □
2.4	**Notes.** Summarise the authors conclusions. Add any comments on your own assessment of the study, and the extent to which it answers your question and mention any areas of uncertainty raised above.	

SIGN	**Methodology Checklist 2: Controlled Trials**		
Study identification (*Include author, title, year of publication, journal title, pages*)			
Guideline topic:		Key Question No:	Reviewer:

Before completing this checklist, consider:

1. Is the paper a **randomised controlled trial** or a **controlled clinical trial**? If in doubt, check the study design algorithm available from SIGN and make sure you have the correct checklist. If it is a **controlled clinical trial** questions 1.2, 1.3, and 1.4 are not relevant, and the study cannot be rated higher than 1+
2. Is the paper relevant to key question? Analyse using PICO (Patient or Population Intervention Comparison Outcome). IF NO REJECT (give reason below). IF YES complete the checklist.

Reason for rejection: 1. Paper not relevant to key question ☐ 2. Other reason ☐ (please specify):

Section 1: Internal validity			
In a well conducted RCT study…		***Does this study do it?***	
1.1	The study addresses an appropriate and clearly focused question	Yes ☐<br>Can't say ☐	No ☐
1.2	The assignment of subjects to treatment groups is randomised.	Yes ☐<br>Can't say ☐	No ☐
1.3	*An adequate concealment method is used.*	Yes ☐<br>Can't say ☐	No ☐
1.4	The design keeps subjects and investigators 'blind' about treatment allocation.	Yes ☐<br>Can't say ☐	No ☐
1.5	The treatment and control groups are similar at the start of the trial.	Yes ☐<br>Can't say ☐	No ☐
1.6	The only difference between groups is the treatment under investigation.	Yes ☐<br>Can't say ☐	No ☐
1.7	All relevant outcomes are measured in a standard, valid and reliable way.	Yes ☐<br>Can't say ☐	No ☐
1.8	What percentage of the individuals or clusters recruited into each treatment arm of the study dropped out before the study was completed?		
1.9	*All the subjects are analysed in the groups to which they were randomly allocated (often referred to as intention to treat analysis).*	Yes ☐<br>Can't say ☐	No ☐<br>Does not apply ☐
1.10	Where the study is carried out at more than one site, results are comparable for all sites.	Yes ☐<br>Can't say ☐	No ☐<br>Does not apply ☐

SECTION 2: OVERALL ASSESSMENT OF THE STUDY		
2.1	How well was the study done to minimise bias?<br>*Code as follows:*	High quality (++)☐<br>Acceptable (+)☐<br>Low quality (-)☐<br>Unacceptable – reject 0 ☐
2.2	Taking into account clinical considerations, your evaluation of the methodology used, and the statistical power of the study, are you certain that the overall effect is due to the study intervention?	
2.3	Are the results of this study directly applicable to the patient group targeted by this guideline?	
2.4	**Notes.** Summarise the authors' conclusions. Add any comments on your own assessment of the study, and the extent to which it answers your question and mention any areas of uncertainty raised above.	

Methodological quality of included studies using STROBE and CONSORT checklist, by study

Studies included	STROBE score	CONSORT score
**Burgraaf, 2001**	52%	-
**Cacciatori, 1996**	61%	-
**Cai, 2018**	70%	-
**Chen, 2006**	66%	-
**Chen, 2007**	53%	-
**Chen, 2010**	63%	-
**Eustatia-Rutten, 2008**	-	60%
**Falcone, 2014**	61%	-
**Galetta, 2009**	53%	-
**Goichot, 2004**	44%	-
**Kabir, 2009**	56%	-
**Karthik, 2009**	53%	-
**Mavai, 2018**	53%	-
**Ngassam, 2018**	59%	-
**Osman, 2004**	47%	-
**Peixoto de Miranda, 2018**	64%	-
**Petretta, 2001**	81%	-
**Pitzalis, 1998**	53%	-
**Portella, 2007**	50%	-
**Ramanathan, 2014**	47%	-
**Tobaldini, 2008**	34%	-
**Tudoran, 2019**	53%	-

Figure S3. Characteristics of included studies

Study	Country	Design	Subgroup	Untreated hyperthyroidism						Healthy controls			ECG, min	HRV parameters
				n	Age, years	Sex, %men	FT4, pmol/L	FT3, pmol/L	TSH, mIU/L	n	Age, years	Sex, %men		
**Burggraaf 2001**	The Netherlands	Prospective	Overt	14	38.9±9.7	7.1%	64.8±18.9	-	0.2±0.3	14	39.5±10.3	7.1%	20	RR, SDNN, TP, LF, HF, LF/HF
**Cacciatori 1996**	Italy	Prospective	Lying – overt	10	39.3±4.5	10.0%	41.0±3.0	-	<0.01	10	38.5±4.2	10.0%	10	RR, TP, LF, HF, LF/HF
			Standing – overt	10	39.3±4.5	10.0%	41.0±3.0	-	<0.01					
			Lying – overt before treatment	8	-	-	-	-	<0.01					
			Standing – overt before treatment	8	-	-	-	-	<0.01					
			Lying – overt before propanolol	4	-	-	-	-	-					
			Standing – overt before propanolol	4	-	-	-	-	-					
**Cai 2018**	China	Prospective	Overt	57	35.0±13.0	38.6%	122±104	28.6±4.9	<0.01	55	39.0±14.0	49.1%	1440	RR, SDNN, RMSSD, pNN50, LF, HF, VLF, LF/HF
**Chen 2006**	Taiwan	Prospective	Overt	32	31.0±2.0	9.4%	72.9±3.1	30.5±1.1	<0.01	32	30.0±1.0	9.4%	30	RR, TP, LF, HF, VLF, LF/HF
**Chen 2007**	Taiwan	Prospective	Overt	33	31.0±1.0	9.1%	69.1±2.8	29.7±1.1	<0.01	33	30.0±1.0	9.1%	30	RR, SDNN
**Chen 2010**	Taiwan	Cross-sectional	Overt	36	30.0±1.0	11.1%	71.6±2.8	30.2±1.0	<0.01	36	29.0±1.0	11.1%	30	RR, SDNN, TP, LF, HF, VLF, LF/HF
**Eustatia-Rutten 2008**	The Netherlands	Randomised controls trial	Subclinical no treatment	13	49.0±7.2	30.8%	22.8±4.3	3.4±0.6	0.1±0.1	15	40.0±10.3	6.7%	15	RR, SDNN
			Subclinical before treatment	12	51.0±10.5	33.3%	22.6±3.9	3.6±0.4	0.1±0.1					
			Overt	15	39.0±9.7	6.7%	-	-	-					
**Falcone 2014**	Italy	Cross-sectional	Subclinical	28	67.0±14.1	17.9%	15.4±7.3	4.6±1.8	0.2±0.1	170	71.0±12.4	34.7%	1440	RR, SDNN, RMSSD, pNN50
**Galetta 2010**	Italy	Cross-sectional	Subclinical	30	49.6±7.6	16.7%	12.7±0.5	5.8±0.3	0.1±0.02	30	50.4±6.8	16.7%	1440	SDNN, RMSSD, pNN50, LF, HF, LF/HF
**Goichot 2004**	France	Cross-sectional	Overt	19	38.6±3.2	15.8%	48.2±5.0	19.0±2.0	<0.01	32	42.9±2.9	43.8%	35	RR, SDNN, LF, HF
			Subclinical	12	49.5±2.7	50.0%	18.6±0.4	5.8±0.2	0.04±0.01					
**Kabir 2009**	Bangladesh	Cross-sectional	Overt	30	38.9±2.4	-	51.4±7.6	-	0.02±0.01	20	39.2±3.6	-	5	RR, SDNN, RMSSD
**Karthik 2009**	India	Cross-sectional	Overt	15	33.1±9.6	0.0%	30.9±11.1	16.1±2.3	0.1±0.02	15	27.8±6.6	0.0%	4	RR, SDNN, RMSSD, TP, LF, HF, LF/HF
**Mavai 2018**	India	Cross-sectional	Overt	35	34.4±8.2	-	175±82.3	12.1±9.7	0.1±0.1	25	34.5±10.1	-	5	SDNN, RMSSD, pNN50, TP, LF, HF, LF/HF
**Ngassam 2018**	Cameroon	Cross-sectional	Subclinical	10	40.3±10.9	10.0%	-	-	-	10	40.9±11.9	10.0%	1440	RR, SDNN, RMSSD, LF, HF, LF/HF
**Osman 2004**	United Kingdom	Prospective	Overt	224	49.0±12.5	23.2%	35.8±12.0	11.3±5.0	-	435	50.0±14.0	26.7%	1440	RR, SDNN, RMSSD, pNN50
			Subclinical	110	49.0±12.5	16.4%	16.4±2.0	5.5±0.5	-					
**Peixoto de Miranda 2018**	Brazil	Retrospective	Subclinical	136	51.0±6.5	37.5%	16.2±5.7	-	0.3±0.1	8623	50.0±6.0	48.4%	10	RR, SDNN, RMSSD, pNN50, LF, HF
**Petretta 2001**	Italy	Cross-sectional	Overt	30	49.0±10.0	20.0%	34.8±7.3	9.4±1.2	<0.01	20	47.0±9.0	20.0%	1440	RR, SDNN, RMSSD, pNN50, LF, HF, VLF, LF/HF
			Subclinical	30	48.0±10.0	16.7%	16.7±4.9	4.5±0.6	0.05±0.03					
**Pitzalis 1998**	Italy	Cross-sectional	Overt	10	37.0±2.8	33.3%	40.9±6.6	15.4±1.7	<0.01	10	37.0±2.1	33.3%	1440	RR, TP, HF
**Portella 2007**	Brazil	Cross-sectional	Subclinical	16	40.0±6.0	0.0%	17.6±3.5	-	0.03±0.07	16	34.5±5.0	0.0%	5	HF, LF/HF
**Ramanathan 2014**	India	Cross-sectional	Overt	30	39.2±6.9	-	-	-	-	30	42.1±6.8	-	10	RR, SDNN, RMSSD, pNN50, LF, HF, VLF, LF/HF
**Tobaldini 2008**	Italy	Cross-sectional	Subclinical	12	-	0.0%	-	-	-	9	-	0.0%	5	RR, HF
**Tudoran 2019**	Romania	Cross-sectional	Severe overt	34	34.7±6.2	0.0%	50.4±12.6	28.1±4.6	<0.01	29	38.9±7.5	0.0%	1440	SDNN, RMSSD, TP, LF, HF, LF/HF
			Moderate overt	46	39.6±5.9	0.0%	25.5±8.3	18.3±6.6	0.03±0.03					
			Chronic overt	33	39.9±6.6	0.0%	48.0±18.6	26.9±3.4	0.01±0.02					

FT4: free thyroxine, FT3: free triiodothyronine, TSH: thyroid-stimulating hormone, RR: RR intervals (or normal-to-normal intervals-NNs), SDNN: standard deviation of RR intervals, pNN50: percentage of adjacent NN intervals differing by more than 50 milliseconds, RMSSD: the square root of the mean squared difference of successive RR-intervals, TP: total power, LF: low frequency, HF: high frequency, VLF: very low frequency, LF/HF ratio: low frequency / high frequency ratio

Figure S3. Study designs of included studies, Aims of included articles, Quality of articles, Inclusion and exclusion criteria of included studies, Characteristics of population, Characteristics of hyperthyroidism, and HRV measurements and analysis

1. Aims of included articles

All included articles aimed to compare HRV between patients with untreated hyperthyroidism and controls without hyperthyroidism [37–58]. Six studies compared HRV parameters in untreated hyperthyroid patients and controls at rest and after vagal and/or sympathetic stimulation [38, 46, 50, 54, 55, 57]. Two studies assessed HRV parameters depending on the severity of hyperthyroidism [46, 58]. Eight studies compared hyperthyroid patients with controls before treatment [37–40, 43, 44, 47, 51].

2. Quality of articles

Using SIGN, mean quality score was 53.9 ± 10.0% for cohort studies, ranging from 31.3% [58] to 68.8% [39, 51] for Yes responses, and the score was 80.0% for the RCT [43] (Figure S2). Few studies were at high level of proof mainly due to the lack of blind to exposure status. Scores were 55.9 ± 9.7% for observational studies using STROBE, ranging from 34.4% [57] to 81.3% [52], and was 60.0% for the RCT using CONSORT [43]. Overall, the studies performed worst in the methods section (Figure S2).

3. Inclusion and exclusion criteria of included studies

All studies included untreated hyperthyroid patients. Most articles included newly diagnosed patients [37–42, 47–50, 54, 56, 57], except one that included patients with hyperthyroidism ≥10 years [43]. Other studies did not report duration of hyperthyroidism [44–46, 51–53, 55, 58]. Most studies included patients regardless of their age, sex or BMI, except few studies that included only patients between 20-60 years [49] or 30-50 years [47, 56, 57], women [48, 55, 57, 58], or with a BMI <25 kg/m² [57] or between 17.3-23 kg/m² [38]. The main exclusion criteria were pregnancy [37, 40–42, 44, 47], severe graves' ophtalmopathy [37, 39], chronic heart [37–45, 47, 49, 50, 52–55, 57, 58], liver [37, 45, 48, 49, 55] or renal [37, 44, 45, 47, 49, 55] failure, use of chronic medications [37, 38, 40–42, 48, 57] or treatment influencing HRV parameters [39, 43, 45, 46, 49, 51, 52, 55, 56, 58] or thyroid status [45, 47, 52, 55], hyperthyroid heart disease [39, 50], thyroid cancer [39], diabetes mellitus [39–42, 45, 47–49, 53, 54, 56–58], hypertension [39, 43, 45, 47–49, 53, 56, 58], cardiac arrhythmia [39–42, 50, 53, 54, 58] and smokers [47, 57].

All studies included controls without hyperthyroidism [37–58], without any coronary artery disease [44] or without any disease [37–43, 45–58]. In each individual study, exclusion criteria were the same as for hyperthyroidism. Healthy controls were paired with untreated hyperthyroid patients based on age [37, 38, 41, 42, 45, 46, 48–50, 54, 56, 57], sex [37, 38, 40–42, 45–48, 50, 54, 57], BMI [38, 51, 52] and body weight [37].

4. Characteristics of population

Sample size ranged from 20 [38, 50, 54] to 8759 [52], for a total of 10811 patients: 1002 with untreated hyperthyroidism and 9809 healthy controls.

Age was reported in all studies except one [57]. The mean age of hyperthyroid patients was 39.3 ± 3.1 years, ranging from 30 [42] to 67 years [44], and 38.0 ± 2.9 years in the controls, ranging from 28 [48] to 71 years [44].

Sex was reported in all studies except three [47, 49, 56]. The proportion of men was 19 ± 6%, ranging from 7% [37] to 50% [46] in hyperthyroid patients, and 22 ± 10% in the controls, ranging from 7% [37, 43] to 48% [52]. Four studies included only women [48, 55, 57, 58].

Body mass index was 22.5 ± 1.3 kg/m² in hyperthyroid patients, ranging from 17.4 [49] to 26.5 kg/m² [55] and 23.1 ± 1.1 kg/m² in controls, ranging from 20.1 [38] to 26.3 kg/m² [58]. BMI was not reported in six studies [43, 46, 51, 53, 54, 57].

Blood pressure was 126/76 ± 6.2/4.0 mmHg in hyperthyroid patients, ranging from 122[39]/65[48] to 133[45]/85[53] mmHg, and 120/76 ± 3.8/5.5 mmHg in controls, ranging from 104[48]/68[40] to 128/81[45]mmHg. Blood pressure was not reported in fifteen [37, 40–44, 46, 47, 49, 51, 52, 55–58].

Heart rate was 81.7 ± 6.3 beats per minute (bpm) in hyperthyroid patients, ranging from 68.8 [52] to 106.7 [47] bpm, and 69.2 ± 3.7 bpm in controls, ranging from 63.5 [58] to 77.0 [48] bpm. Heart rate was not reported in thirteen studies [37–43, 46, 49, 53–55, 57].

Other characteristics were seldomly reported such as *metabolic parameters* (blood glucose levels, total cholesterol, and triglycerides) [39, 45], *rhythm disorders* [45, 51, 58], and *smoking* [44, 45, 52, 55].

5. Characteristics of hyperthyroidism

Thyroid function was described clinically and biologically in all studies. Twelve studies included overt hyperthyroidism [37–42, 47–49, 54, 56, 58], six subclinical [44, 45, 50, 52, 55, 57] and four both [43, 46, 51, 53]. Several articles did not report fT3 [37, 38, 47, 50, 52, 55–57], TSH [50, 51, 56, 57] and fT4 levels [50, 56, 57] in hyperthyroid patients. In controls, the proportion increased to 68% for FT3 levels [37, 38, 40–43, 47, 48, 50–52, 54–57], and 45% for both fT4 and TSH [38, 40–43, 48, 50, 54, 56, 57]. Mean ft4 levels were 36.9 ± 6.7 pmol/L in hyperthyroid patients, ranging from 12.7 [45] to 122.3 [39] pmol/L, and 14.9 ± 1.1 pmol/L in controls, ranging from 10.8 [47] to 18.0 [39] pmol/L. Mean ft3 levels were 15.5 ± 3.1 pmol/L in hyperthyroid patients, ranging from 3.4 [43] to 30.5 [40] pmol/L, and 5.3 ± 0.4 pmol/L in controls, ranging from 4.0 [49] to 5.8 [45] pmol/L. TSH levels were 0.01 ± 0.01 mIU/L in hyperthyroid patients, ranging from undetectable [38, 46, 54] to 0.30 [52] mIU/L, and 1.93 ± 0.31 mIU/L in controls, ranging from 1.37 [55] to 4.00 [39] mIU/L.

Etiologies of hyperthyroidism were mainly Graves' disease [37, 38, 40–42, 44, 46, 53, 54], from 20% [53] to 100% [37, 38, 40–42] of patients, then toxic multinodular goiter [44–46, 53–55] affecting 10% [45, 54] to 27% [53] of patients, and toxic adenoma [44–46, 53, 55], from 5% [46] to 50% [53]. Iatrogenic causes [43, 44, 55] were rarer. Others studies did not specify the etiology of hyperthyroidism [39, 47, 49–52, 56–58].

Duration of hyperthyroidism was reported in 14 studies. Most studies included newly diagnosed and untreated hyperthyroid patients before initiation of the treatment [37–42, 47–50, 54, 56, 57], except one study that included patients with at least 10 years of hyperthyroidism [43]. Others studies did not indicate a specific duration of hyperthyroidism, but patients remained untreated [44–46, 51–53, 55, 58].

6. HRV measurements and analysis

Recording of HRV measurements was ambulatory, spontaneous breathing with normal daily activity in all studies. Most studies used ECG, achieved in a resting supine position, to determine HRV [37, 38, 43, 46–49, 52, 55–57] between four [48] and 30 minutes [40–42], except eight studies using a 24-hour holter-ECG [39, 44, 45, 50, 51, 53, 54, 58]. Nearly all studies had a distinct Holter monitoring system: Cambridge Electronic Design model 1401 [37, 43], Life Scoope 6 [38], Marquette 3000 [39], Cardisuny 501B [40], Pathfinder 700 series analyser [44, 51], Diagnostic monitoring system [45], Colin PressMate 8800 [48], RMS ECG [49], Polar RS 800 [50], Teac-Tascam 234 Syncaset [53], Micromed [52], ECG Hewlett Packard 78354C [54], Biopotentials Captation System [55], Niviqure digital ECG system [56] and Holter Labtech Cardiospy [58]. Six studies did not report the monitoring system [41, 42, 46, 47, 57]. Premature atrial and ventricular beats were automatically discarded and visually checked.

Parameters reported were both time and frequency domains in most studies, except five studies that reported only time domain [41, 43, 44, 47, 51] and one only frequency domain [55]. For time domain parameters, RR intervals was reported in 18 studies [37–44, 46–48, 50–54, 56, 57], SDNN in 16 studies [37, 39, 41–49, 51–53, 56, 58], RMSSD in 12 studies [39, 44, 45, 47–53, 56, 58], pNN50 in 8 studies [39, 44,

45, 49, 51–53, 56]. For frequency domain parameters, TP was reported in 8 articles [37, 38, 40, 42, 48, 49, 54, 58], LF power in 12 studies [37–40, 42, 45, 46, 49, 52, 53, 56, 58], LFnu in 9 studies [38–40, 42, 46, 48–50, 56], HF power in 12 studies [37–40, 42, 45, 46, 49, 52, 53, 56, 58], HFnu in 10 studies [38–40, 42, 48–50, 54–56], VLF in 5 studies [39, 40, 42, 53, 56] and LF/HF in 13 studies [37–40, 42, 45, 48–50, 53, 55, 56, 58]. All stratifications depending on severity of hyperthyroidism had more than four studies, except for LFnu, HFnu, VLF and TP in subclinical hyperthyroidism. To measure frequency domain parameters, two main methods were used: Fast Fourier transform model in six studies [37, 40, 42, 45, 46, 53], and autoregressive model in four studies [38, 52, 54, 57]. Seven studies did not specify the method used [39, 48–50, 55, 56, 58]. Other parameters or methods of measure were seldomly reported such as heart rate turbulence parameters [51, 58], corrected QT interval dispersion [39, 45], or corrected conditional entropy [57] and nonlinear methods [41, 42].

Figure S5. Detailed meta–analyses in untreated hyperthyroid patients compared with controls for each HRV parameters: RR intervals, SDNN, RMSSD, pNN50, TP, LF, HF, VLF, LF/HF

RR: RR intervals (or normal-to-normal intervals-NNs), SMD : standardised mean differences(effect size)

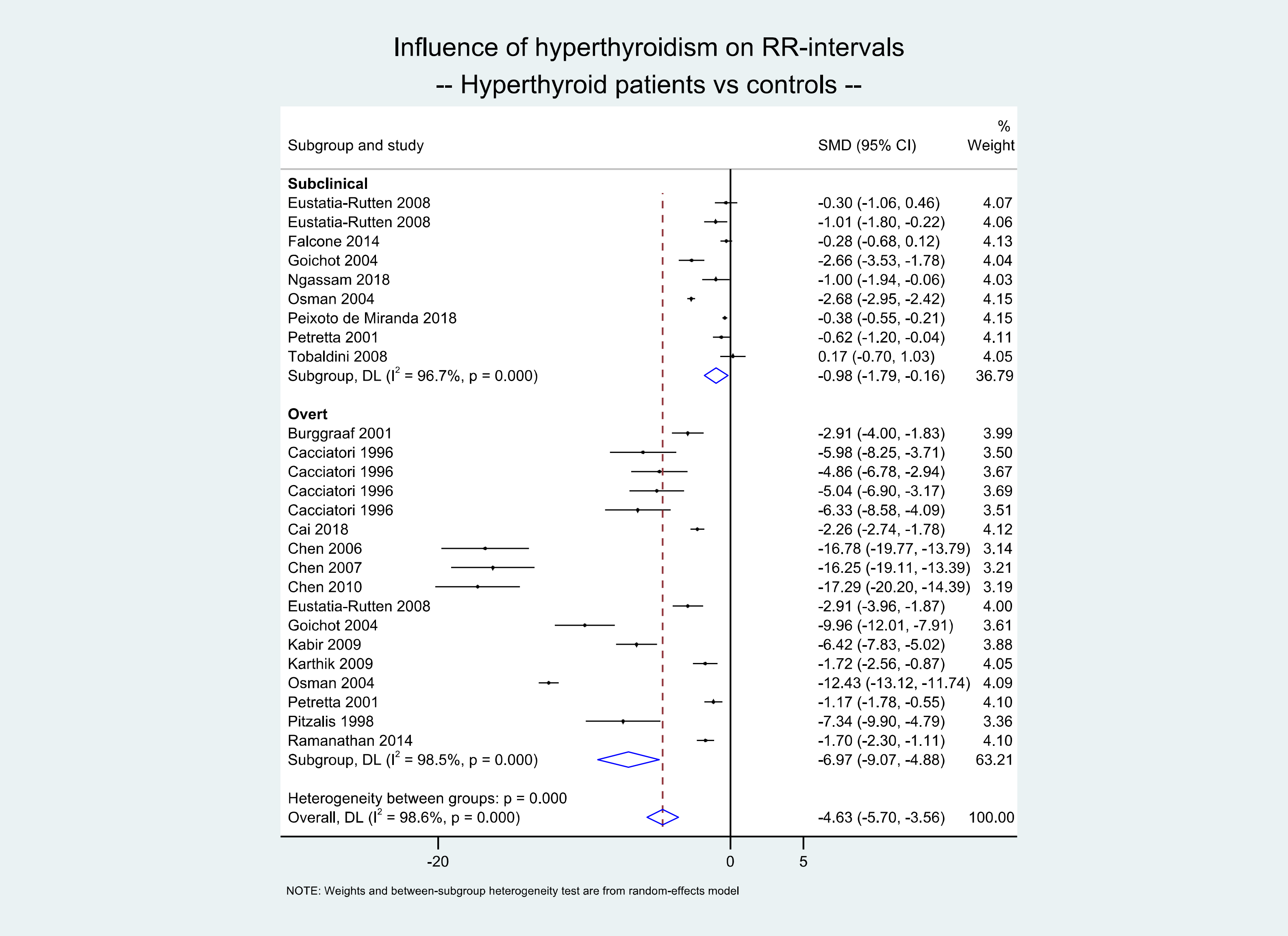

SDNN: standard deviation of RR intervals, SMD : standardised mean differences (effect size)

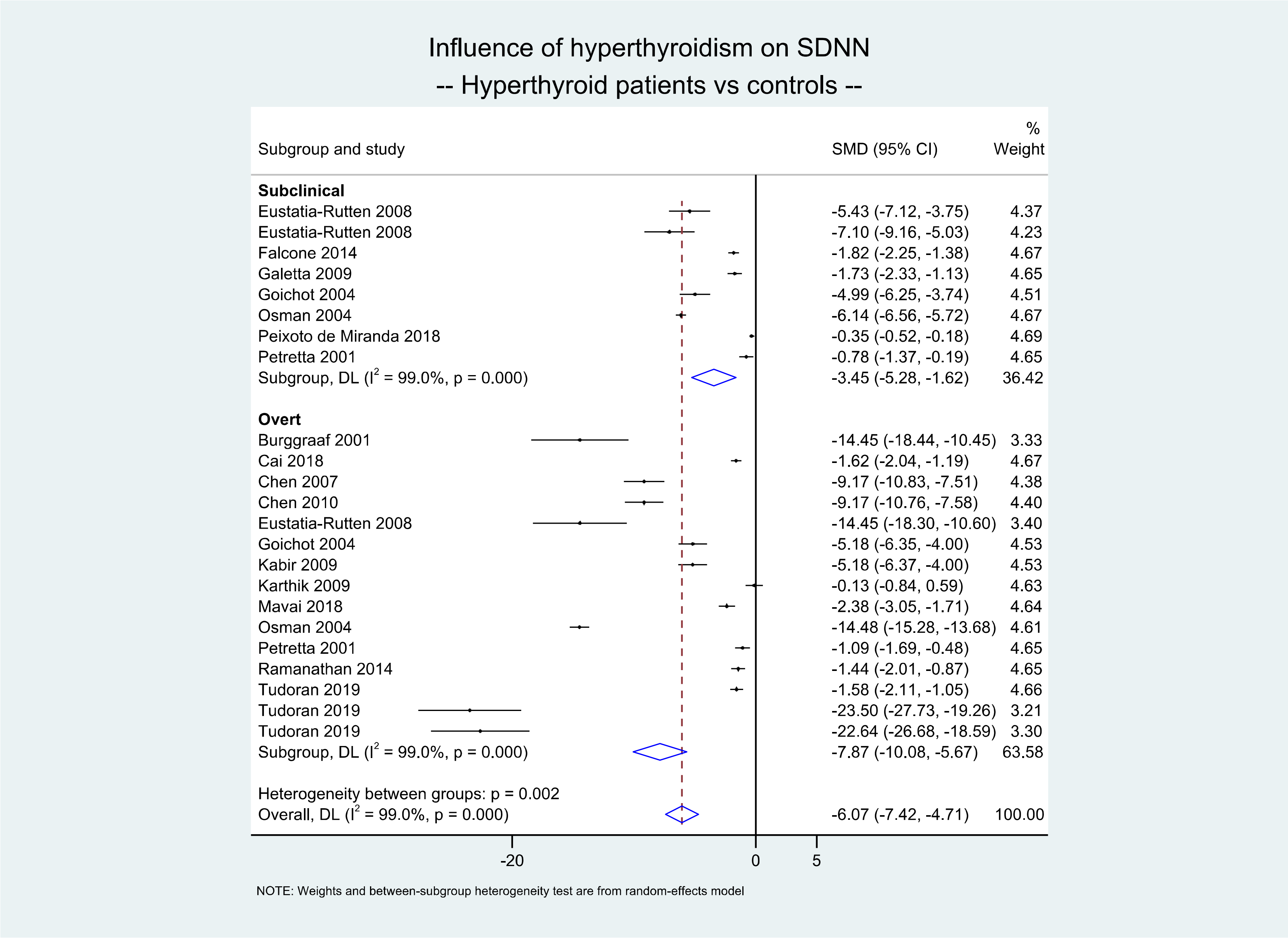

RMSSD: the square root of the mean squared difference of successive RR-intervals SMD : standardised mean differences (effect size)

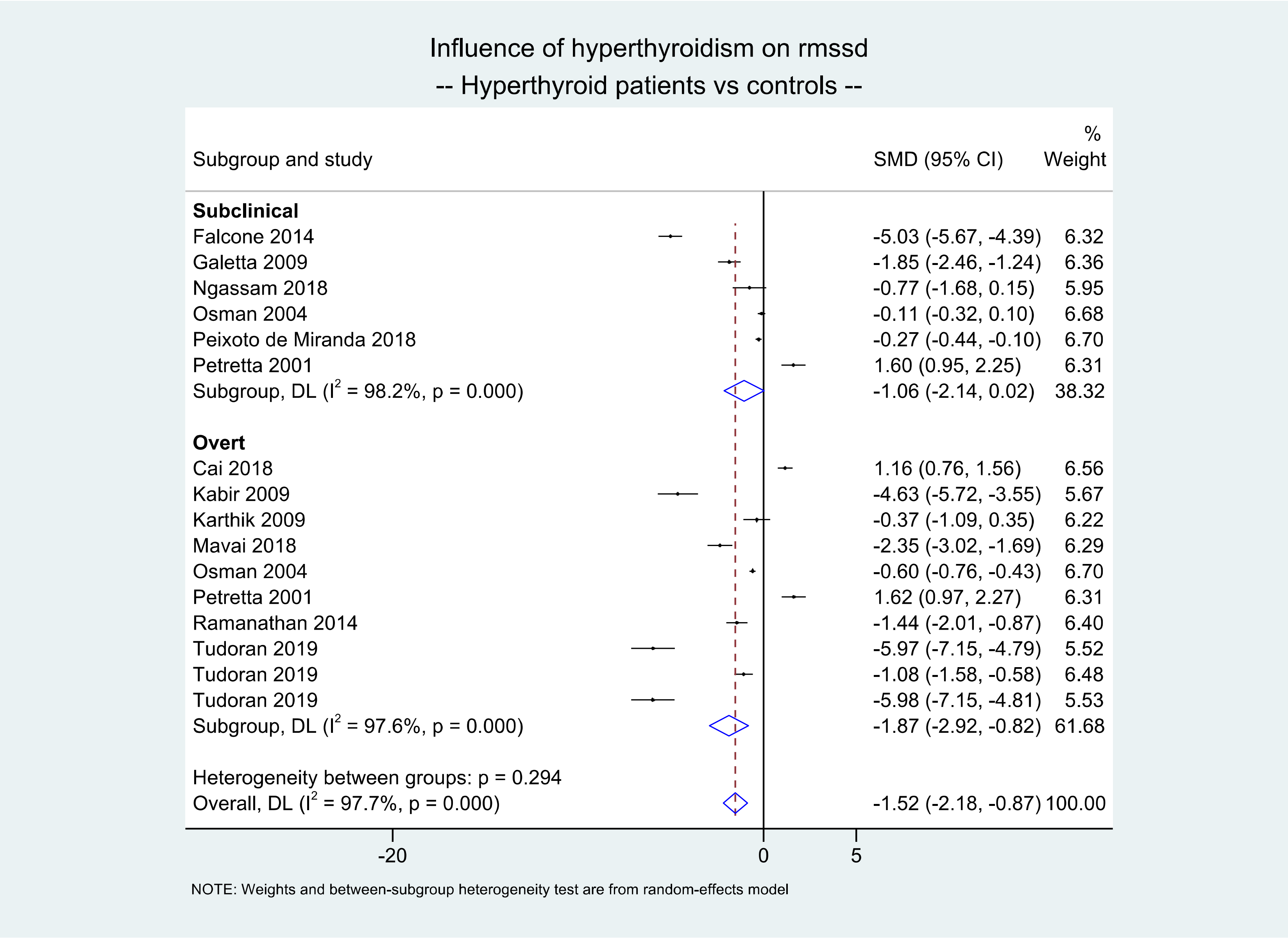

pNN50 : percentage of adjacent NN intervals differing by more than 50 milliseconds, SMD : standardised mean differences (effect size)

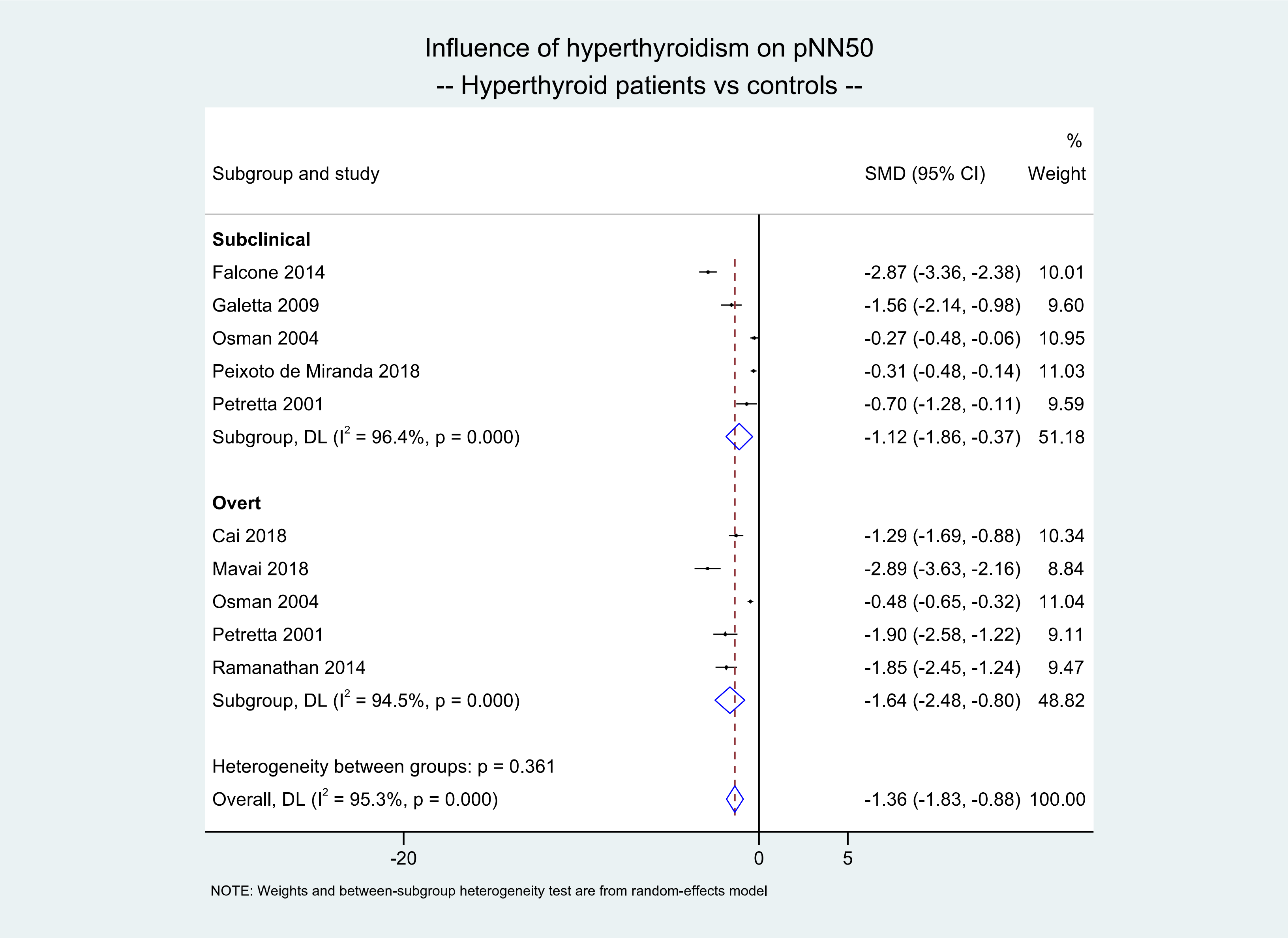

SMD: standardised mean differences (effect size)

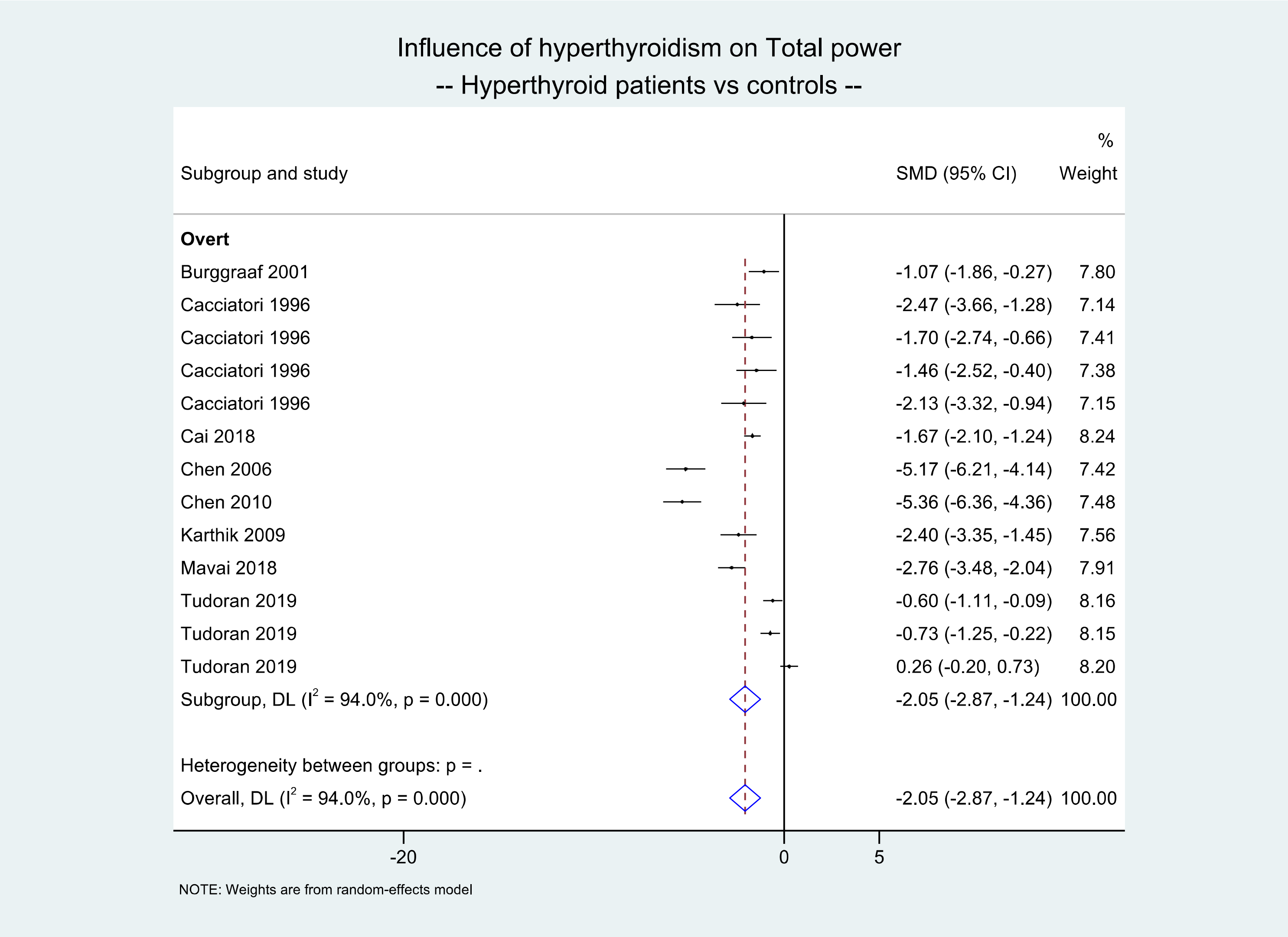

LF: low frequency, LFnu: low frequency normalized – units, SMD : standardised mean differences(effect size)

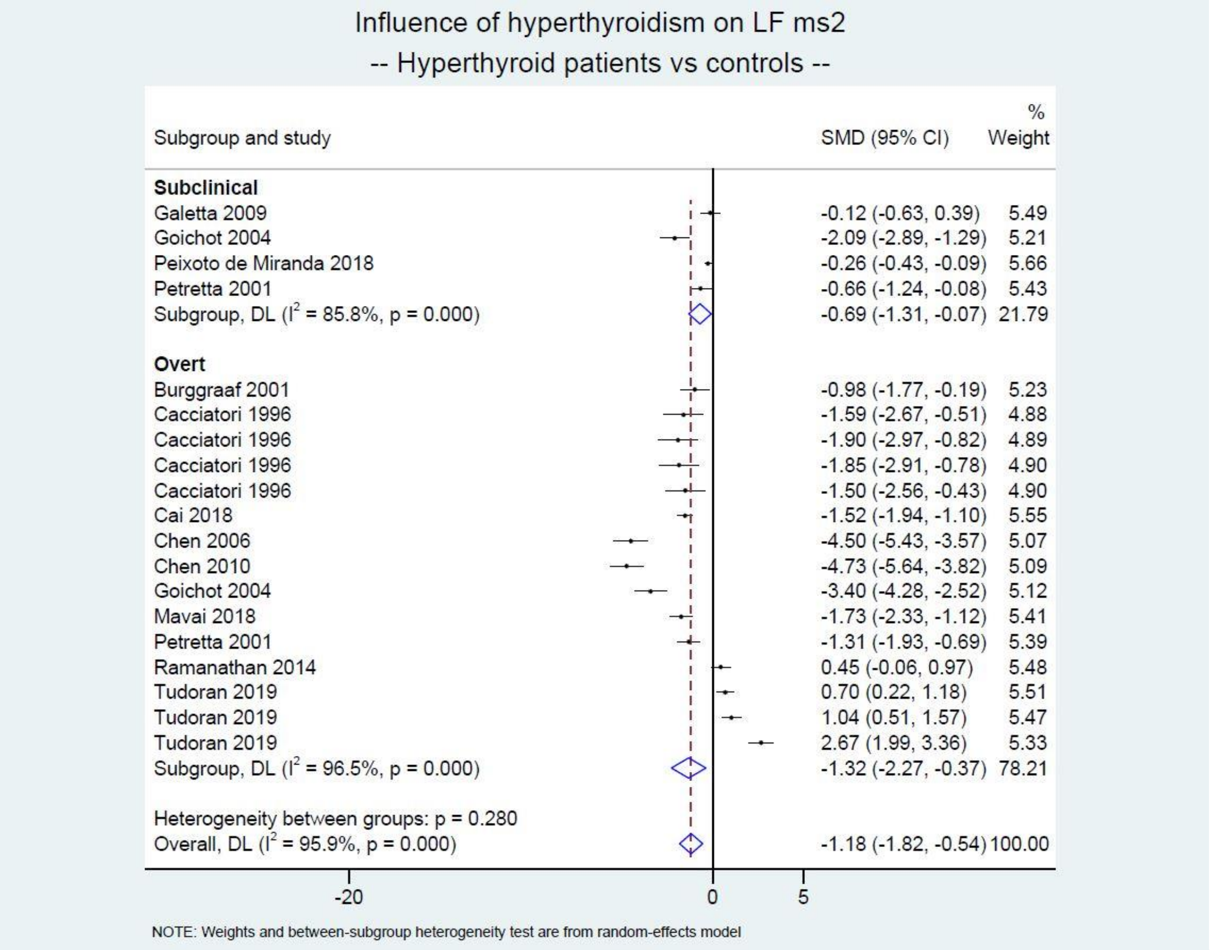

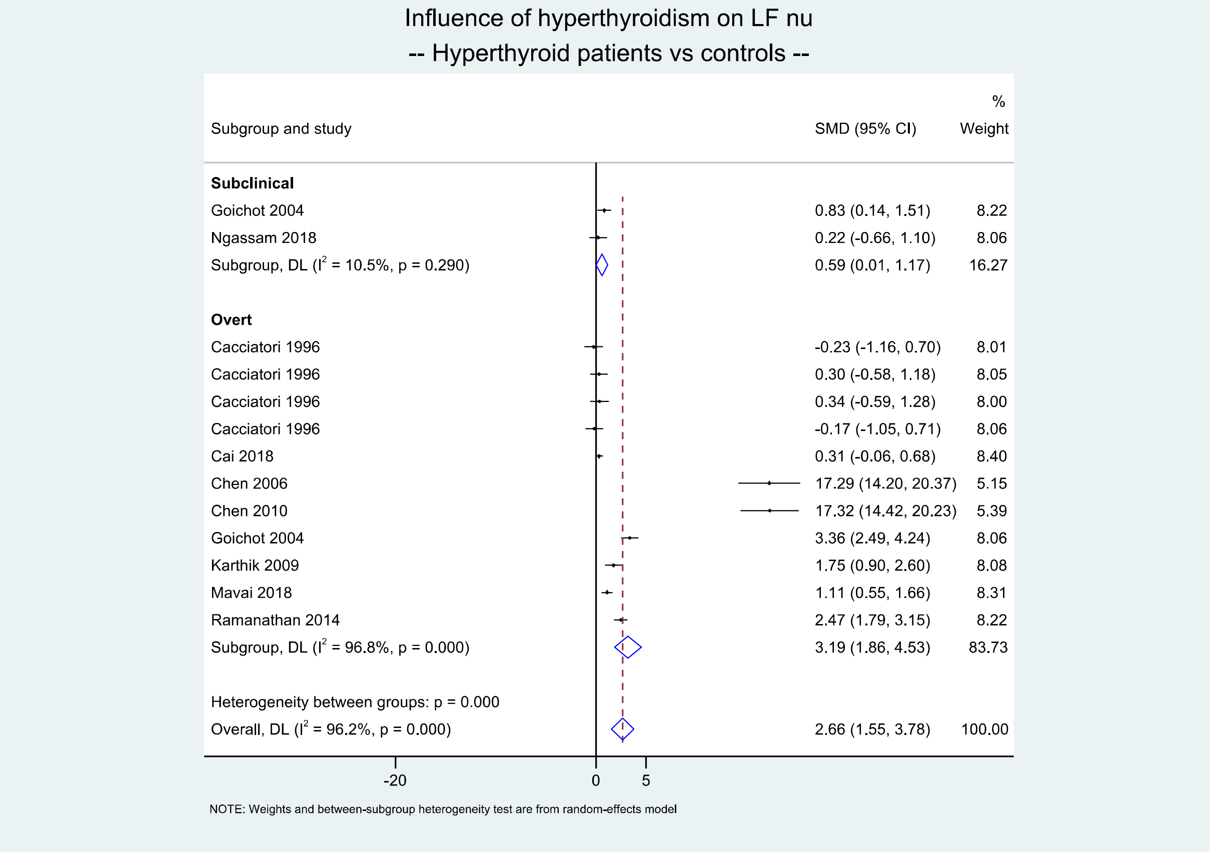

HF: high frequency, HFnu: high frequency – normalized units, SMD : standardised mean differences(effect size)

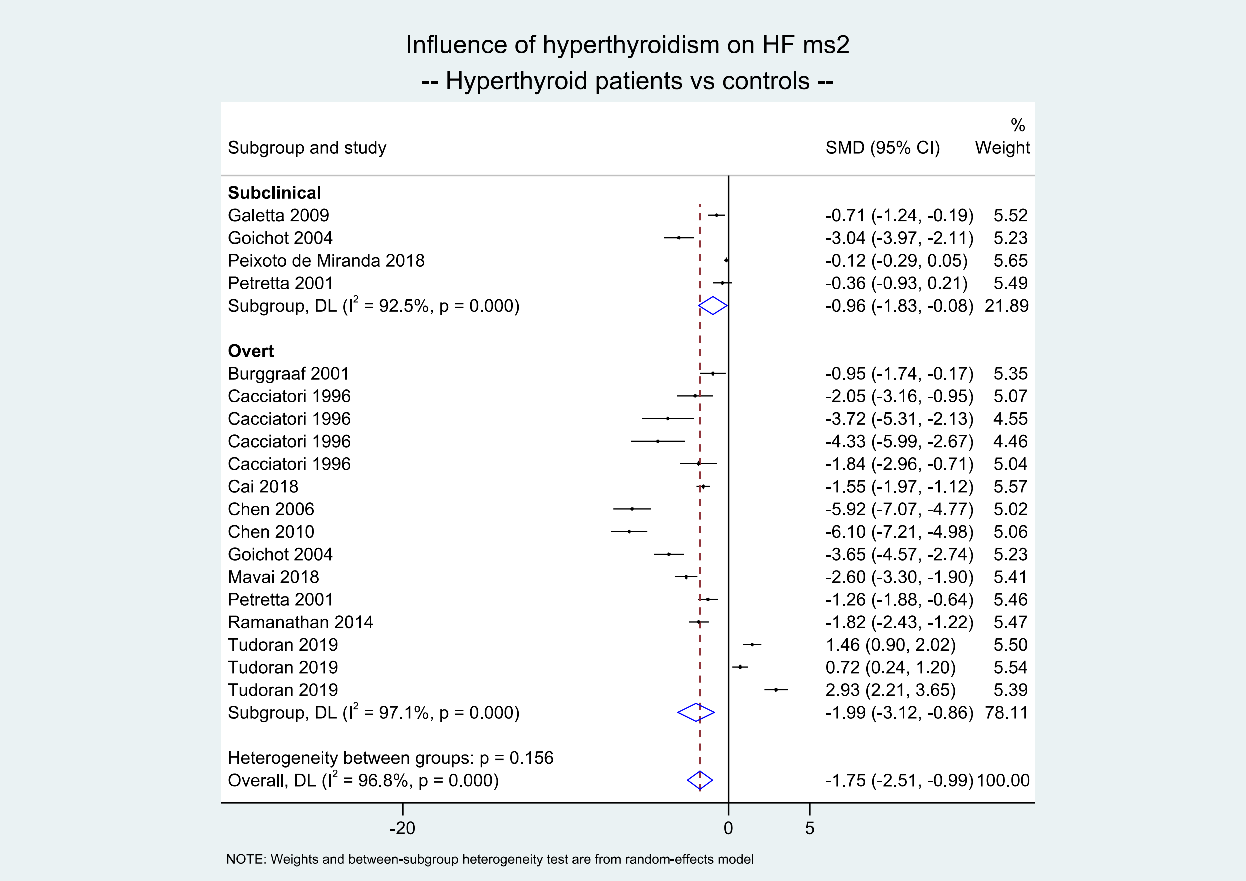

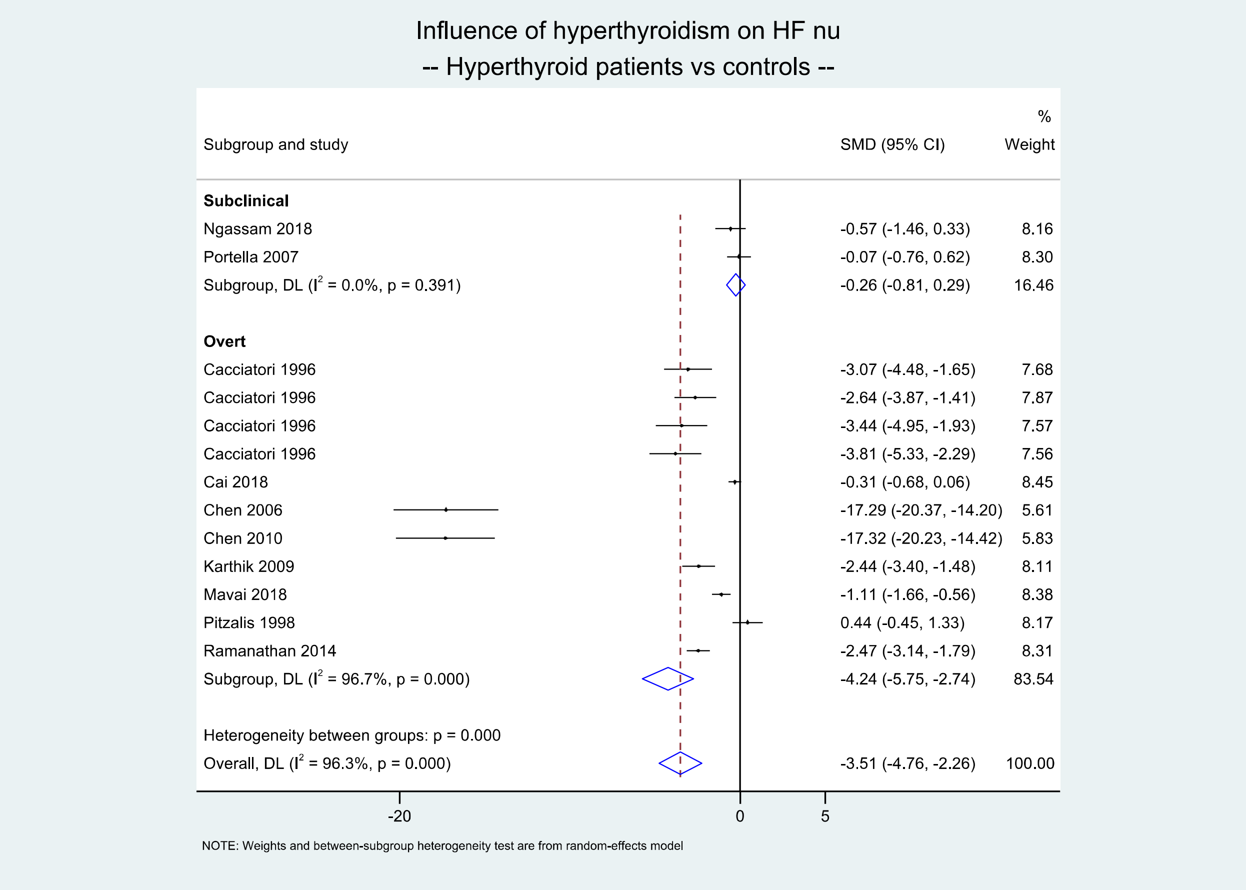

VLF: very low frequency, SMD: standardised mean differences (effect size)

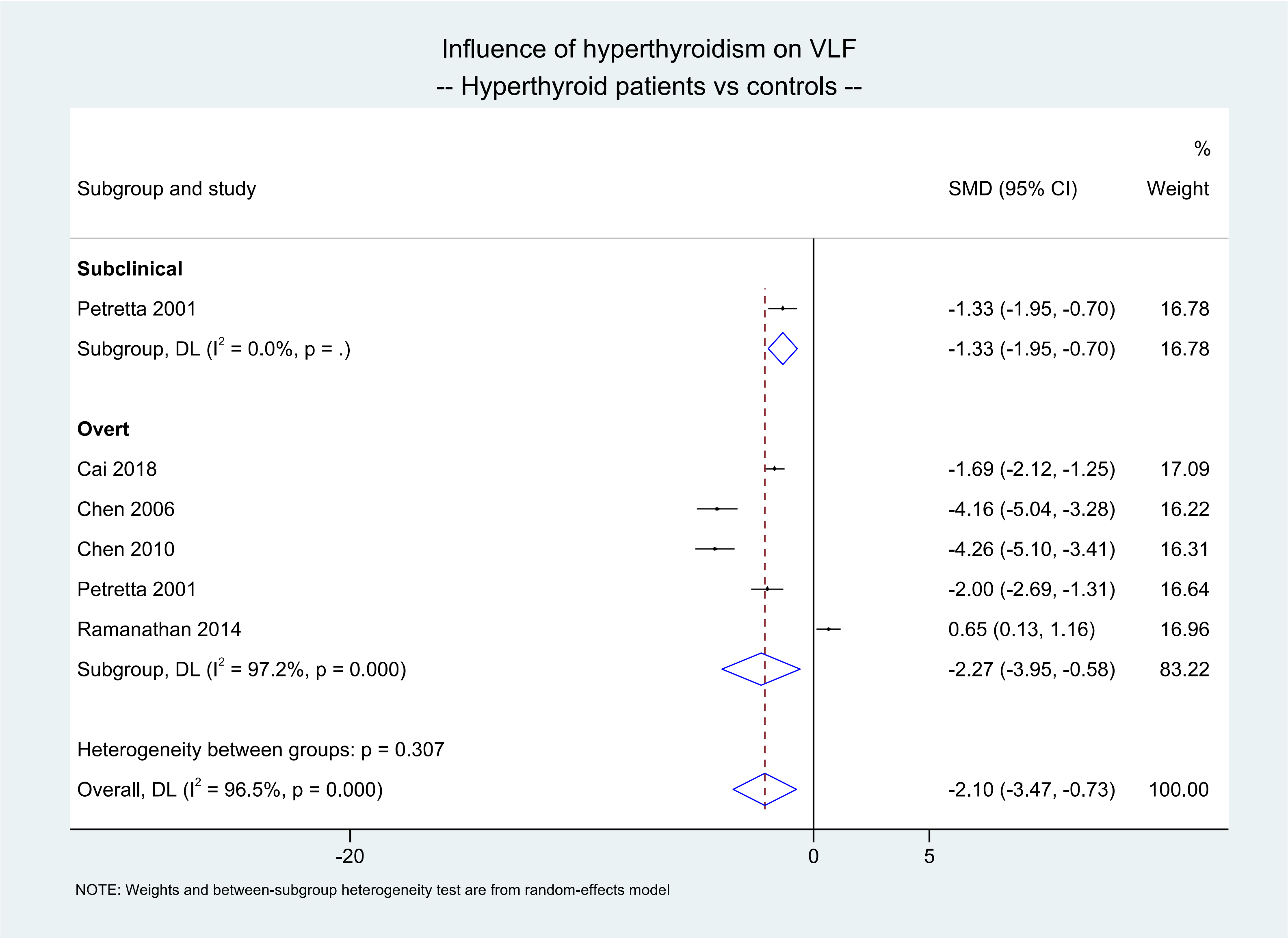

LF/HF ratio: low frequency / high frequency ratio, SMD : standardised mean differences(effect size)

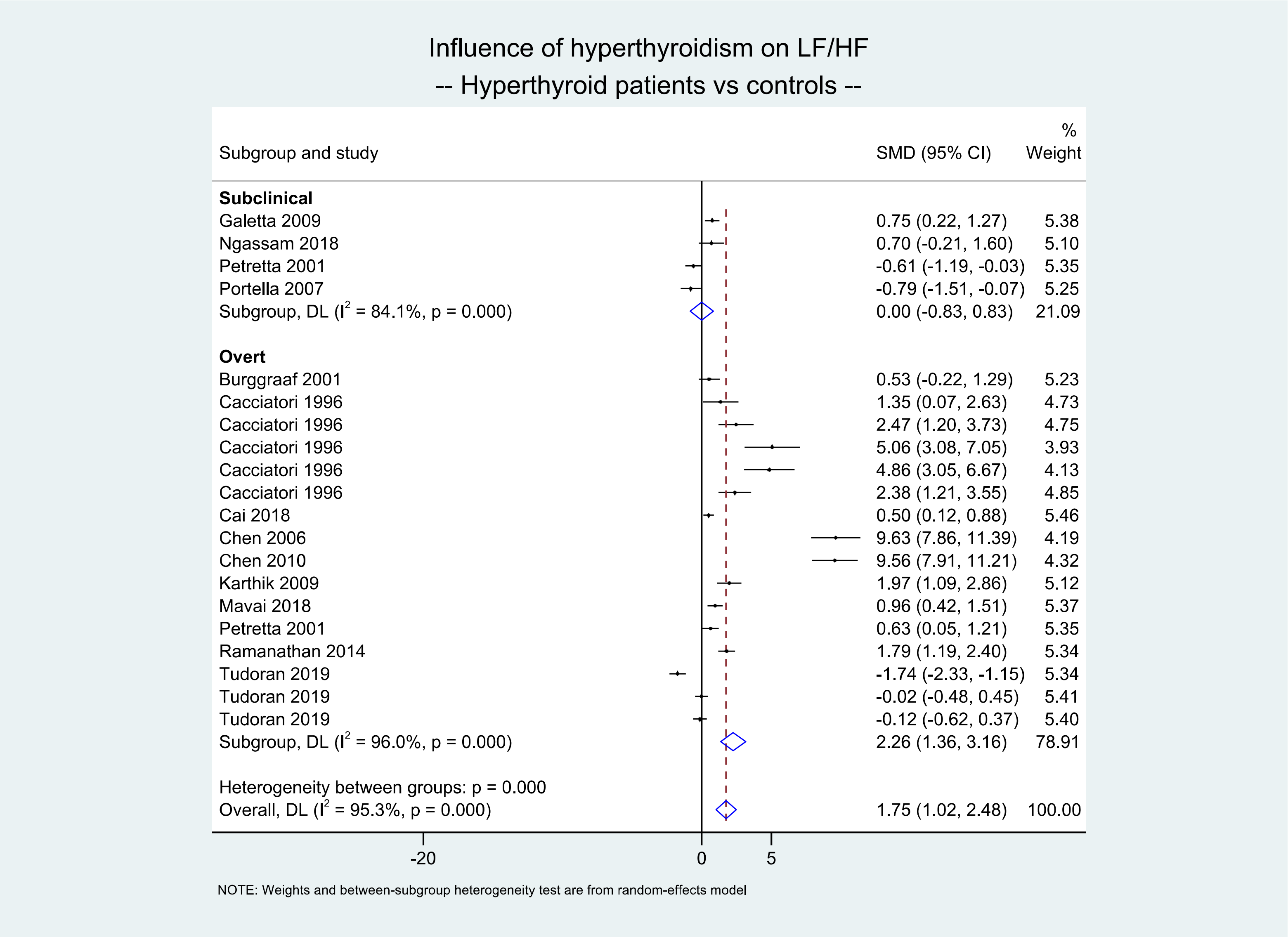

Figure S6. Detailed meta-regressions of factors influencing HRV parameters

	n subgroups	I-squared (%)	Coefficient (95% CI)		p-value
**RR**					
Subclinical vs overt	26	98.2%	-6.00	(-9.75 to -2.25)	**0.003**
Age, years	25	98.6%	0.35	(0.13 to 0.58)	**0.003**
Sex, %men	22	98.9%	0.05	(-0.19 to 0.31)	0.68
BMI, kg/m²	13	96.0%	1.69	(0.03 to 3.35)	**0.046**
SBP, mmHg	8	91.6%	0.10	(-0.94 to 1.13)	0.82
DBP, mmHg	8	90.5%	0.13	(-0.19 to 0.46)	0.36
FT4, pmol/L	20	98.9%	-0.10	(-0.19 to -0.01)	**0.034**
FT3, pmol/L	15	99.0%	-0.47	(-0.71 to -0.22)	**0.001**
TSH, mUI/L	18	96.8%	35.7	(2.53 to 68.9)	**0.037**
**SDNN**					
Subclinical vs overt	23	99.0%	-4.63	(-10.5 to 1.24)	0.12
Age, years	23	99.1%	0.20	(-0.14 to 0.53)	0.23
Sex, %men	20	99.2%	0.05	(-0.26 to 0.36)	0.75
BMI, kg/m²	14	97.6%	-0.67	(-2.31 to 0.96)	0.39
SBP, mmHg	5	82.6%	0.01	(-0.24 to 0.25)	0.95
DBP, mmHg	5	75.9%	-0.05	(-0.17 to 0.07)	0.25
FT4, pmol/L	21	99.1%	-0.01	(-0.09 to 0.07)	0.78
FT3, pmol/L	18	99.0%	-0.31	(-0.63 to 0.01)	0.06
TSH, mUI/L	19	97.0%	23.5	(-20.9 to 67.9)	0.28
**RMSSD**					
Subclinical vs overt	16	97.8%	-0.85	(-3.68 to 1.98)	0.53
Age, years	16	97.8%	0.00	(-0.16 to 0.17)	0.96
Sex, %men	13	97.8%	10.1	(-1.27 to 21.5)	0.08
BMI, kg/m²	12	98.1%	-0.10	(-0.65 to 0.46)	0.70
SBP, mmHg	6	75.1%	-0.30	(-0.49 to -0.09)	**0.015**
DBP, mmHg	6	95.8%	0.07	(-0.15 to 0.29)	0.44
FT4, pmol/L	14	98.1%	0.00	(-0.04 to 0.03)	0.89
FT3, pmol/L	12	98.3%	-0.10	(-0.32 to 0.09)	0.27
TSH, mUI/L	12	98.4%	1.92	(-21.1 to 24.9)	0.86
**pNN50**					
Subclinical vs overt	10	95.7%	-0.52	(-1.99 to 0.94)	0.44
Age, years	10	95.9%	0.00	(-0.09 to 0.08)	0.96
Sex, %men	8	95.6%	2.46	(-6.94 to 11.9)	0.55
BMI, kg/m²	6	95.4%	0.17	(-0.23 to 0.57)	0.29
SBP, mmHg	4	70.2%	-0.03	(-0.32 to 0.25)	0.65
DBP, mmHg	4	71.3%	-0.07	(-0.54 to 0.42)	0.59
FT4, pmol/L	9	95.0%	-0.01	(-0.02 to 0.01)	0.22
FT3, pmol/L	8	96.2%	-0.01	(-0.13 to 0.12)	0.92
TSH, mUI/L	7	93.9%	2.89	(-7.33 to 13.1)	0.50
**VLF**					
Subclinical vs overt	6	97.2%	-0.94	(-7.09 to 5.21)	0.69
Age, years	6	96.8%	0.12	(-0.14 to 0.39)	0.26
Sex, %men	5	91.5%	7.79	(-8.71 to 24.3)	0.23
BMI, kg/m²	4	0.0%	2.70	(1.73 to 3.68)	**0.007**
SBP, mmHg	3	45.2%	-0.12	(-3.76 to 3.52)	0.75
DBP, mmHg	3	47.5%	-0.03	(-1.09 to 1.03)	0.79
FT4, pmol/L	5	94.6%	-0.01	(-0.07 to 0.05)	0.73
FT3, pmol/L	5	92.9%	-0.08	(-0.22 to 0.07)	0.19
TSH, mUI/L	5	93.7%	32.4	(-78.3 to 143.1)	0.42

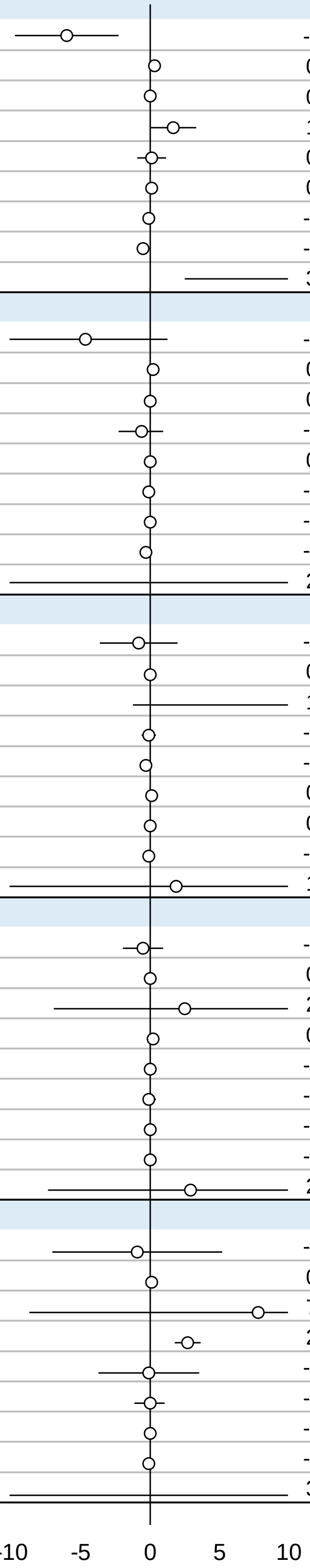

RR: RR intervals (or normal-to-normal intervals-NNs), BMI: Body mass index, SBP: systolic blood pressure, DBP: diastolic blood pressure, FT4: free thyroxine, FT3: free triiodothyronine, TSH: thyroid-stimulating hormone, SDNN: standard deviation of RR intervals, RMSSD: the square root of the mean squared difference of successive RR-intervals, pNN50: percentage of adjacent NN intervals differing by more than 50 milliseconds, VLF: very low frequency

	n subgroups	I-squared (%)	Coefficient (95%CI)		p-value
**Total power**					
Age, years	13	88.8%	0.35	(0.15 to 0.56)	**0.003**
Sex, %men	10	95.2%	-3.40	(-16.1 to 9.22)	0.55
BMI, kg/m²	11	89.1%	0.41	(0.07 to 0.76)	**0.023**
SBP, mmHg	4	0.0%	-0.09	(-0.38 to 0.19)	0.29
DBP, mmHg	4	0.0%	0.06	(-0.11 to 0.22)	0.28
FT4, pmol/L	11	94.3%	-0.01	(-0.04 to 0.02)	0.40
FT3, pmol/L	8	96.8%	-0.10	(-0.39 to 0.19)	0.43
TSH, mUI/L	11	95.5%	8.31	(-20.5 to 37.2)	0.53
**LF**					
Subclinical vs overt	19	96.0%	-0.55	(-2.73 to 1.63)	0.60
Age, years	19	95.8%	0.09	(-0.05 to 0.23)	0.19
Sex, %men	15	96.6%	-0.19	(-0.11 to 0.07)	0.67
BMI, kg/m²	13	94.7%	0.53	(0.19 to 0.86)	**0.005**
SBP, mmHg	6	51.3%	0.12	(-0.02 to 0.26)	0.08
DBP, mmHg	6	73.3%	0.09	(-0.06 to 0.24)	0.16
FT4, pmol/L	16	96.0%	-0.01	(-0.04 to 0.01)	0.32
FT3, pmol/L	12	97.4%	-0.03	(-0.18 to 0.12)	0.67
TSH, mUI/L	16	96.4%	5.29	(-8.15 to 18.7)	0.41
**LFnu**					
Subclinical vs overt	13	96.4%	3.33	(-7.22 to 13.9)	0.50
Age, years	13	96.4%	-0.75	(-1.38 to -0.12)	**0.024**
Sex, %men	9	97.5%	-0.08	(-0.46 to 0.31)	0.62
BMI, kg/m²	9	97.5%	-0.29	(-4.13 to 3.55)	0.86
SBP, mmHg	5	40.1%	0.18	(-0.09 to 0.44)	0.12
DBP, mmHg	5	63.3%	-0.08	(-0.29 to 0.13)	0.30
FT4, pmol/L	9	97.5%	0.00	(-0.12 to 0.13)	0.96
FT3, pmol/L	7	98.2%	0.56	(-0.12 to 1.24)	0.09
TSH, mUI/L	9	97.5%	-75.4	(-317.9 to 167.2)	0.49
**HF**					
Subclinical vs overt	19	96.8%	-0.95	(-3.72 to 1.82)	0.48
Age, years	19	96.6%	0.13	(-0.05 to 0.31)	0.14
Sex, %men	15	97.2%	-0.03	(-0.15 to 0.09)	0.58
BMI, kg/m²	13	94.1%	0.72	(0.32 to 1.11)	**0.002**
SBP, mmHg	6	84.9%	0.08	(-0.35 to 0.51)	0.63
DBP, mmHg	6	74.5%	0.17	(-0.04 to 0.38)	0.09
FT4, pmol/L	16	96.8%	-0.02	(-0.05 to 0.02)	0.33
FT3, pmol/L	12	97.9%	-0.04	(-0.22 to 0.15)	0.67
TSH, mUI/L	16	97.1%	7.60	(-9.65 to 24.9)	0.36
**HFnu**					
Subclinical vs overt	13	96.4%	-4.40	(-14.3 to 5.49)	0.35
Age, years	13	96.6%	1.13	(0.35 to 1.91)	**0.008**
Sex, %men	9	97.4%	11.8	(-36.2 to 59.7)	0.58
BMI, kg/m²	10	97.2%	0.71	(-1.52 to 2.91)	0.48
SBP, mmHg	6	89.1%	-0.12	(-0.93 to 0.69)	0.70
DBP, mmHg	6	90.1%	0.04	(-0.45 to 0.54)	0.81
FT4, pmol/L	9	97.5%	0.00	(-0.13 to 0.12)	0.99
FT3, pmol/L	6	98.5%	-0.77	(-1.78 to 0.23)	0.10
TSH, mUI/L	9	97.5%	-0.02	(-168.2 to 325.6)	0.48
**LF/HF**					
Subclinical vs overt	20	95.4%	2.38	(-1.06 to 5.82)	0.16
Age, years	19	95.6%	-0.30	(-0.55 to -0.05)	**0.021**
Sex, %men	15	96.2%	2.11	(-17.5 to 21.7)	0.82
BMI, kg/m²	15	95.3%	-0.69	(-1.28 to -0.08)	**0.031**
SBP, mmHg	8	88.5%	0.02	(-0.43 to 0.43)	0.92
DBP, mmHg	8	85.1%	-0.11	(-0.28 to 0.07)	0.19
FT4, pmol/L	15	96.2%	0.01	(-0.03 to 0.06)	0.55
FT3, pmol/L	11	97.1%	0.18	(-0.08 to 0.43)	0.15
TSH, mUI/L	15	96.4%	-21.7	(-67.1 to 23.6)	0.32

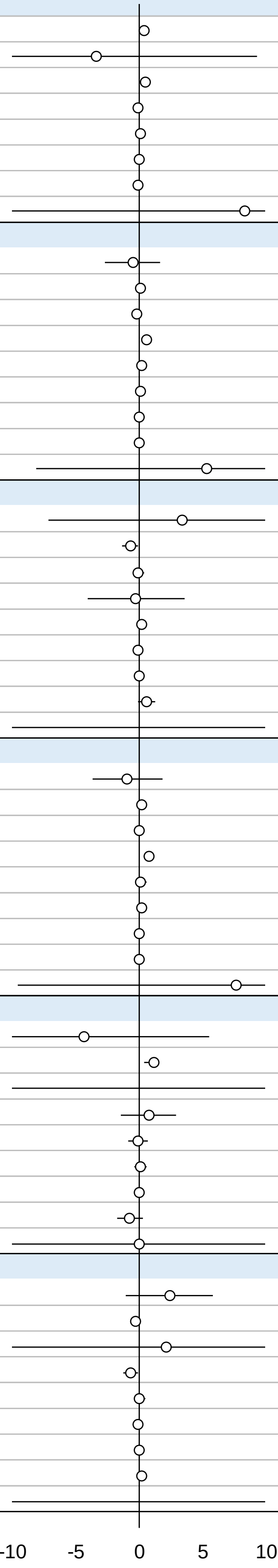

BMI: Body mass index, SBP: systolic blood pressure, DBP: diastolic blood pressure, FT4: free thyroxine, FT3: free triiodothyronine, TSH: thyroid-stimulating hormone LF: low frequency, LFnu: low frequency normalized – units, HF: high frequency, HFnu: high frequency – normalized units, LF/HF ratio: low frequency / high frequency ratio

Figure S7. Metafunnels

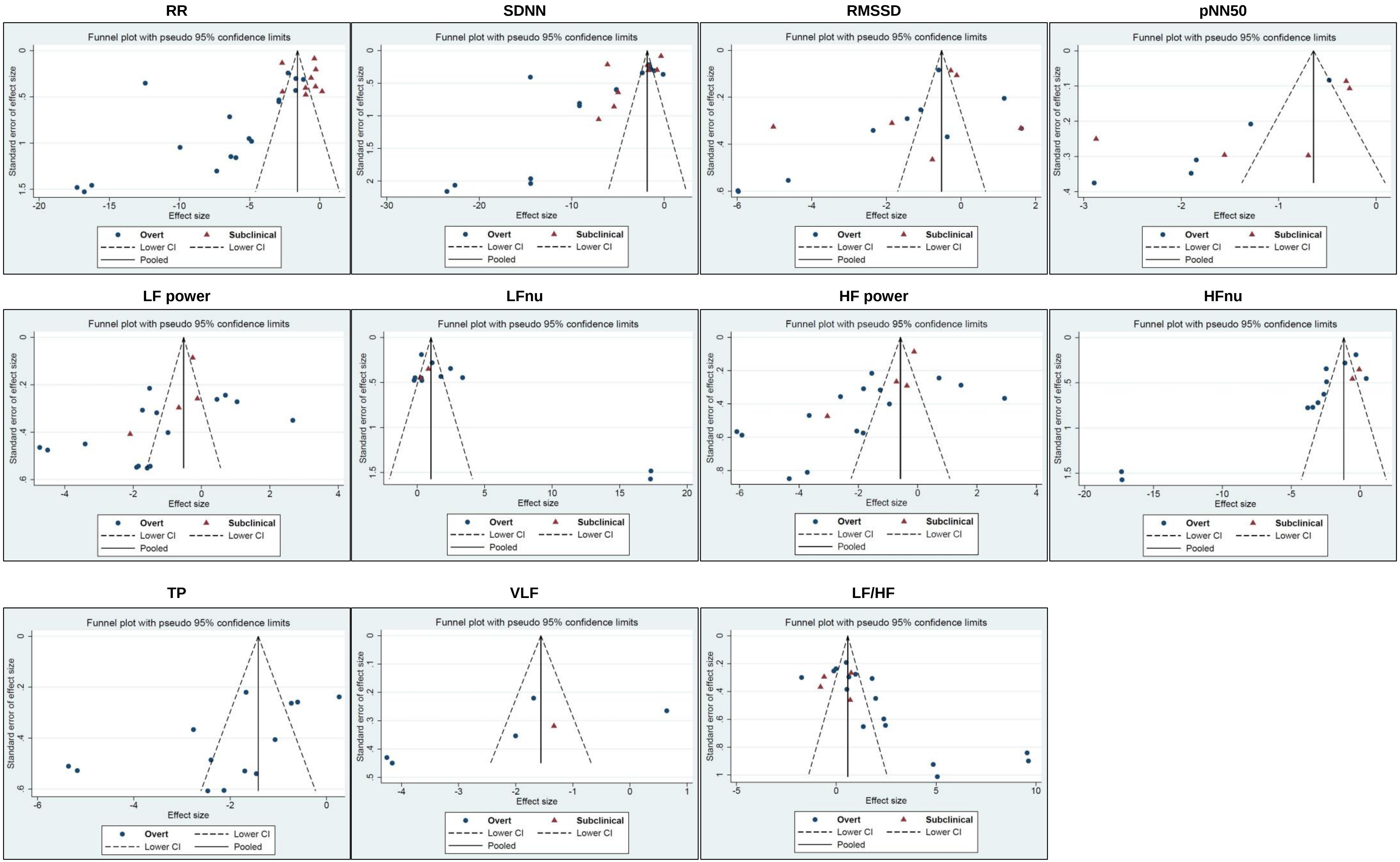

RR: RR intervals (or normal-to-normal intervals-NNs), SDNN: standard deviation of RR intervals, RMSSD: the square root of the mean squared difference of successive RR-intervals, pNN50: percentage of adjacent NN intervals differing by more than 50 milliseconds, LF power: low frequency, LFnu: low frequency normalized – units, HF power: high frequency, HFnu: high frequency – normalized units, TP: total power, VLF: very low frequency, LF/HF ratio: low frequency / high frequency ratio
